# Revision of the southeast Asian soldier-fly genus *Parastratiosphecomyia* Brunetti, 1923 (Diptera, Stratiomyidae, Pachygastrinae)

**DOI:** 10.3897/zookeys.238.3999

**Published:** 2012-11-05

**Authors:** Norman E. Woodley

**Affiliations:** 1 Systematic Entomology Laboratory–PSI–ARS–USDA, Smithsonian Institution NHB–168, P O Box 37012, Washington, DC 20013–7012, USA

**Keywords:** Oriental Region, Pachygastrinae, taxonomy, new species, wasp mimicry

## Abstract

The genus *Parastratiosphecomyia* Brunetti is revised with the description of two new species: *Parastratiosphecomyia freidbergi* **sp. n.** from India and *Parastratiosphecomyia rozkosnyi* **sp. n.** from Laos and Thailand. All four species in the genus are illustrated and a key to species is provided. Type localities of previously described taxa are briefly discussed.

## Introduction

The subfamily Pachygastrinae is a somewhat heterogenous group that is defined by having lost vein M_3_, a loss that has occurred numerous times independently in the Stratiomyidae (Woodley 2001). The Oriental genera *Parastratiosphecomyia* and the similar appearing *Stratiosphecomyia* Brunetti were placed by James (1952) in the tribe Meristomeringini, along with some African genera. Woodley (2010) expressed doubts as to whether or not the two Oriental genera were related to the Afrotropical ones. Only a comprehensive phylogenetic study of the Pachygastrinae will elucidate the relationships within the subfamily and determine if the subfamily is monophyletic.

Brunetti (1923) described the genus *Parastratiosphecomyia* based on a single new species, *Parastratiosphecomyia stratiosphecomyioides* Brunetti from Thailand. Subsequently, Lindner (1954) described a second species, *Parastratiosphecomyia szechuanensis*, from China. Other than these original descriptions, the genus has remained unstudied and few additional specimens have been found in museums. New specimens have come to hand from fairly recent collecting in which two undescribed species have been discovered. It is the purpose of this paper to summarize knowledge of this genus and describe the new species.

As can be surmised by the generic and specific names, species of *Parastratiosphecomyia* are excellent mimics of aculeate wasps, possibly sphecids, but putative models have never been recorded. In *Parastratiosphecomyia* the elongate antennae, darkened wings, and strongly clavate abdomen contribute to its resemblance to wasps. Mimicry of wasps and bees has arisen numerous times in the Stratiomyidae with mimics known from at least six subfamilies. At least some of these mimics also have behavior that contributes to the overall effect, such as fast flight, how they hold their wings at rest, and how they sit on perches when not flying (Woodley, pers. obs.).

## Methods

Specimens have been borrowed from several institutions for which acronyms are given that are used in the specimen data citations:

MMB Department of Entomology, Moravian Museum, Brno, Czech Republic

FSMU Faculty of Science, Masaryk University, Brno, Czech Republic

ZIB Institute of Zoology, Slovak Academy of Sciences, Bratislava, Slovakia

MHPC Martin Hauser Personal Collection, Sacramento, California, USA

MNHN Muséum national d’Histoire naturelle, Paris, France

USNM National Museum of Natural History, Smithsonian Institution, Washington, DC, USA

NBC Naturalis Biodiversity Center, Leiden, Netherlands

SMF Senckenberg Forschungsinstitut und Naturmuseum, Frankfurt-am-Main, Germany

BMNH The Natural History Museum, London, England

ZFMK Zoologisches Forschungsinstitut und Museum Alexander Koenig, Bonn, Germany

Specimens were examined with a Zeiss Stemi SV 11 stereomicroscope. Male terminalia were dissected from specimens relaxed in a humidity chamber for about 24 hours, cleared in hot KOH, neutralized with weak acetic acid, and rinsed with water. The terminalia are preserved in a microvial on the specimen pin.

*Parastratiosphecomyia stratiosphecomyioides* Brunetti, 1923, the type species, is redescribed first in detail. Species that follow are described based on how they differ from *Parastratiosphecomyia stratiosphecomyioides*. This methodology is chosen because the known species are very similar in coloration and morphology and full descriptions of each species would be largely repetitious. Morphological terminology follows that of McAlpine (1981) as modified by Cumming and Wood (2009). Body lengths are given exclusive of antennae.

### Parastratiosphecomyia

Brunetti, 1923

http://species-id.net/wiki/Parastratiosphecomyia

Figs 1 [Fig F2] [Fig F3] [Fig F4] [Fig F5] [Fig F6] [Fig F7] [Fig F8] –30

Parastratiosphecomyia Brunetti 1923: 67. Type species, *Parastratiosphecomyia stratiosphecomyioides* Brunetti, by original designation.

#### Diagnosis.

**Figure 1.**
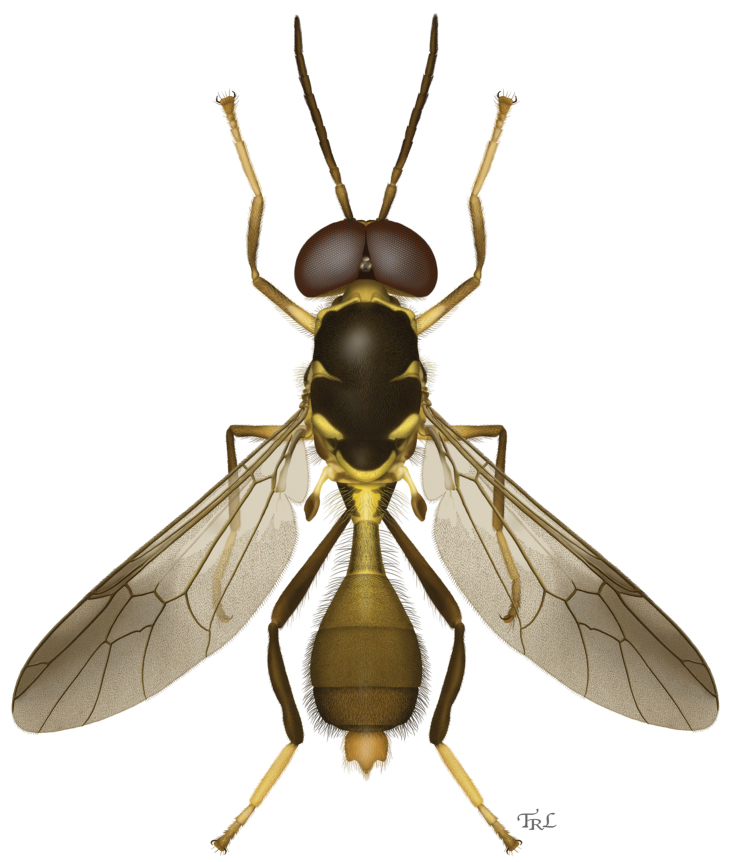
Dorsal habitus illustration of *Parastratiosphecomyia freidbergi* Woodley. Illustration by Taina Litwak.

**Figures 2–4. F2:**
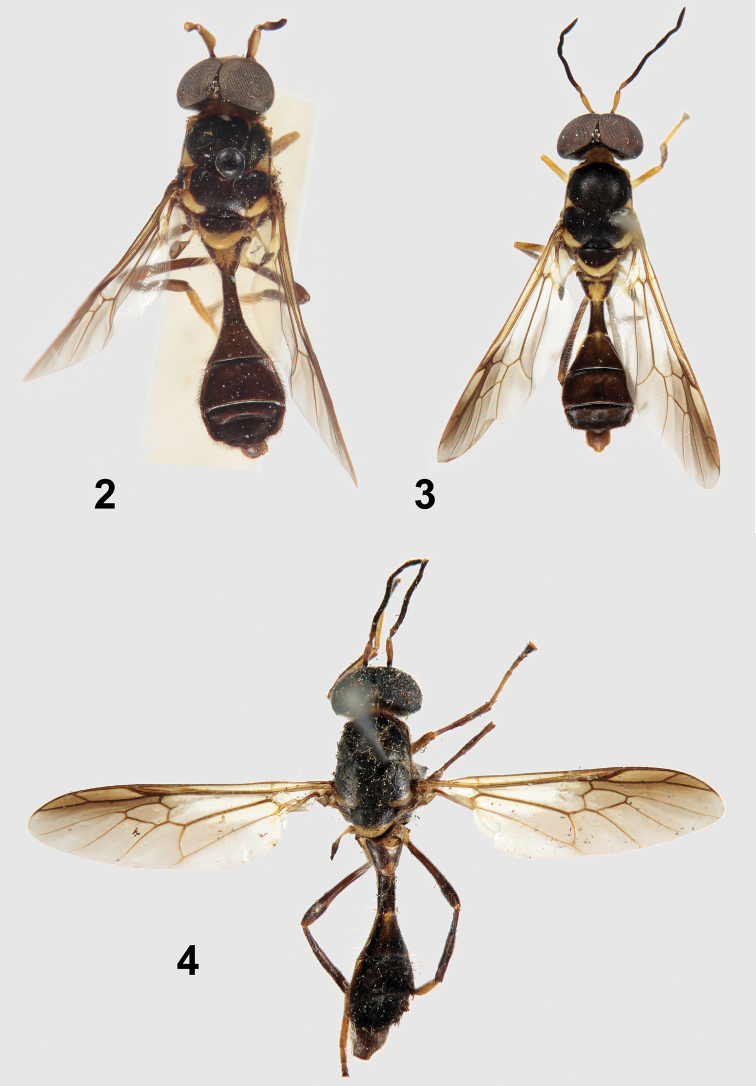
Primary types of *Parastratiosphecomyia* species. **2** *Parastratiosphecomyia sphecomyioides* Brunetti(lectotype) **3** *Parastratiosphecomyia freidbergi* Woodley (holotype) **4** *Parastratiosphecomyia szechuanensis* (holotype) Lindner.

**Figure 5. F3:**
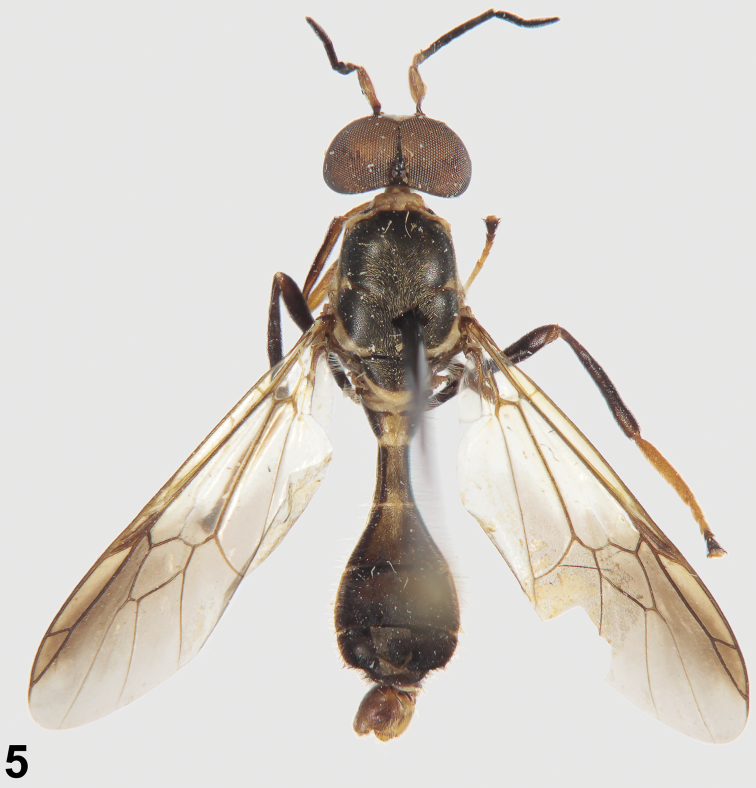
Holotype of *Parastratiosphecomyia rozkosnyi* Woodley.

This genus can be separated from all other genera of Stratiomyidae by its long antennae that have the bases widely separated, the sockets being closer to the eye margin than their diameter (Figs 1–5). This is certainly an apomorphic character state. The pair of bluntly conical processes on the lower face is also an apomorphic feature of this genus (Figs 9, 10).

Slender, elongate, wasp-mimicking flies generally about 10–12 mm in length (Fig. 1). *Head*: Eyes bare, strongly holoptic in males with upper ommatidia enlarged, females with eyes smaller, dichoptic with uniform ommatidia; lower margin of face with a pair of bluntly conical tubercles, each near the intersection of the face with the gena; antenna about three to four times length of head, cylindrical, scape about three times as long as pedicel, flagellum with eight flagellomeres, first six subequal in length, seventh and eighth slightly more elongate; palpus small, two-segmented, nearly cylindrical, second segment two to three times as long as first.

*Thorax*: Scutum convex; scutellum moderately convex, rounded, without spines; post-tegula with some short hairs; legs unmodified, hind femur very slightly clavate, tibiae without spurs; wing infuscated subapically in all known species; mostly set with microtrichia, with noticeable bare areas in cells c, br, bm, and cup and at base of wing; costal vein extending just beyond apex of R_5_, ending before wing apex; R_2+3_ originating beyond r-m by about or slightly more than length of r-m, ending in costa; R_4_ present; discal cell angular, about twice longer than wide; M_3_ absent; crossvein dm-cu absent; alula ovoid, posterior margin rounded, gradually widening distally.

*Abdomen*: Longer than thorax, very strongly clavate, second segment very narrow, almost cylindrical, dorsum of segments 3–5 nearly flat.

#### Remarks.

The four species treated in this revision are very similar in coloration and in morphological details. However, the male terminalia are strikingly different between the species. The terminalia are large and protrude from the end of the abdomen, so some details, particularly the structure of the gonostyli, can be viewed without dissection.

#### Key to the species of *Parastratiosphecomyia*

**Table:** 

1	Antennal scape strongly produced ventrally (Fig. 9); scutellum with apical half or more yellow; Malaysia, Thailand	*Parastratiosphecomyia stratiosphecomyioides*
–	Antennal scape weakly or not modified, not strongly produced ventrally (Fig. 10); scutellum with only margin yellow, the coloration at most one-third of scutellar length	2
2(1)	Hind coxa uniformly pale yellow; tarsi unicolorous, the apical tarsomeres not darker than basal ones; India	*Parastratiosphecomyia freidbergi* sp. n.
–	Hind coxa infuscated on external lateral surface with brownish to blackish coloration; tarsi with apical one or two tarsomeres darkened, brownish to blackish, especially on front and hind legs	3
3(2)	Male with epandrium evenly arcuate in lateral view; gonostylus apically divided into two processes (Fig. 22); female tergite 8 with juncture of posterior and lateral margins evenly curved; Laos, Thailand	*Parastratiosphecomyia rozkosnyi* sp. n.
–	Male with epandrium bent ventrally in lateral view; gonostylus not strongly divided, with a large, sickle-shaped apical lobe (Fig. 27); female tergite 8 with juncture of posterior and lateral margins angulate, produced dorsally with a sharply rounded apex; China, Laos, Vietnam	*Parastratiosphecomyia szechuanensis*

### Parastratiosphecomyia stratiosphecomyioides

Brunetti, 1923

http://species-id.net/wiki/Parastratiosphecomyia_stratiosphecomyioides

Figs 2 , 6 , 9 , 11–15

Parastratiosphecomyia stratiosphecomyioides Brunetti, 1923: 67.

#### Diagnosis.

*Parastratiosphecomyia stratiosphecomyioides* is easily distinguished from the other three known species in the genus by its remarkably produced ventral side of the antennal scape (Fig. 9).

#### Redescription.

**Male.** *Head*: Brownish black, but lower frons and most of face pale yellow, lower frons with small diffuse brownish spots adjacent to inner margins of antennal sockets, face dark near oral margin, including conical processes; face slightly convex but depressed medially on lower part, not concave on lowest part between conical processes, with upper medial portion moderately striate, and with a tiny conical process at lower, outer margin of each antennal socket; lower frons with very narrow band of pale tomentum at eye margins, similar tomentum along eye margin at gena and postgena; face, gena, and ocellar tubercle with short to moderate length silvery white hair-like setae, longest on genal area; antenna with scape and pedicel dark yellow, scape slightly darker dorsally, both with short, dark, semi-appressed hair-like setae, densest dorsally; scape with ventral surface markedly produced, inner region near base with rounded excavation; flagellum blackish with dense pilosity (most of flagellum missing in males examined, presumably as in female described below); palpus with first segment pale yellow, second dark brownish; proboscis dark yellowish.

**Figures 6–8. F4:**
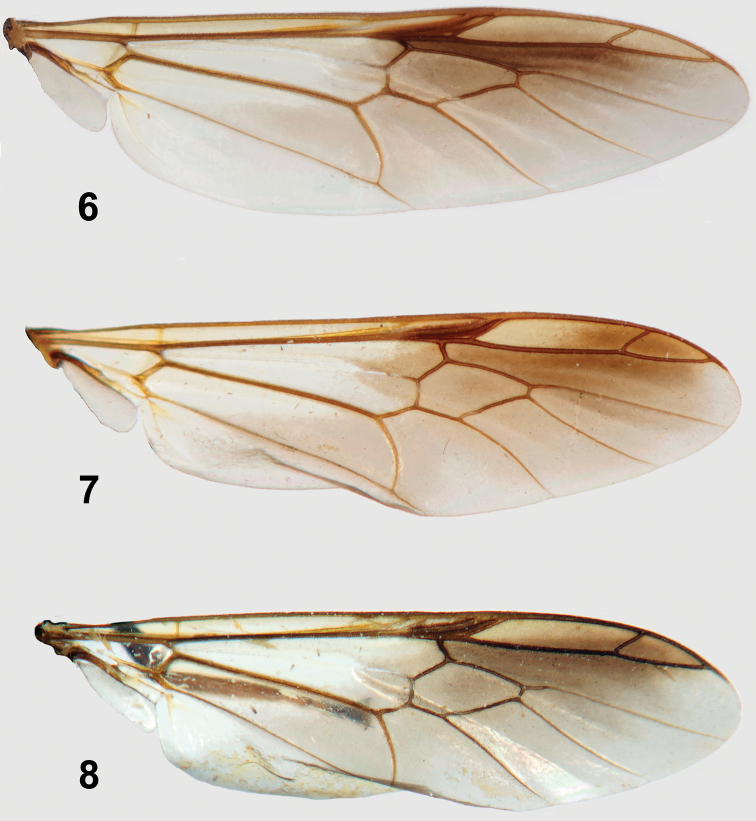
Wings of *Parastratiosphecomyia* species. **6** *Parastratiosphecomyia sphecomyioides* Brunetti **7** *Parastratiosphecomyia freidbergi* Woodley **8** *Parastratiosphecomyia rozkosnyi* Woodley.

*Thorax*: Prothorax yellow but proepisternum is brownish black; scutum black with a pair of triangle-shaped lateral spots that are mostly on the presutural part, and area around postalar callus similarly yellow; scutellum black with apical half or more yellow; pleura yellow but ventral part of anepisternum, most of katepisternum, ventral part of meron, laterotergite and mediotergite brownish black; scutum with short, semi-appressed pilosity consisting of dark hair-like setae on dark cuticular areas, and pale hair-like setae on yellow areas, except laterally slightly longer hair-like setae present that are mostly pale; pilosity of pleura pale, slightly longer than on dorsal part of thorax and more erect, dorsal part of anepisternum bare; legs with coxae and trochanters pale yellow; front femur dark brown at base, gradually becoming dark yellow in basal third, middle and hind femora similar but the basal brown region more extensive, becoming yellow in apical half; front tibia mostly dark yellowish, narrowly dark dorsally; middle and hind tibiae brownish, more yellowish on ventral parts; tarsi yellowish, middle tarsus paler than others; halter with stem yellowish white, knob dark brown; wing (Fig. 6) with subapical cloud of infuscation starting at the proximal edge of discal cell, darkest and most evident in basal two-thirds of cell r_5_.

*Abdomen*: Blackish brown, first tergite with irregular yellowish medial spot, tergites 3-5 with narrow lateral margins vaguely paler; first tergite with moderately long, pale hair-like setae, tergites 2-5 densely set with short, dark hair-like setae on most of dorsal surface, tergites 2-3 with longer pale hair-like setae laterally (similar to those on first segment) and tergites 4-5 with longer dark hairs along lateral and posterolateral margins; sternites 1-3 yellowish, 4-5 brownish, with short, pale hair-like setae basally which become dark from apical half of sternite 3 posteriorly.

*Terminalia*: Gonocoxites (Fig. 11) with lateral margins nearly straight, diverging posteriorly, with a pair of rounded processes posterior to gonocoxal apodemes; gonocoxal apodemes extending anteriorly to about anterior margin of genital capsule; synsternite of genital capsule with posterior process that is slightly bilobed; gonostyli arcuate, without processes (Figs 11, 12); phallic complex (Figs 13, 14) small, trilobed, the medial lobe shorter than lateral lobes; epandrium (Fig. 15) large, evenly convex, slightly indented posterolaterally, posterior margin truncate; hypoproct sclerotized, but not expanded dorsolaterally.

*Length*: 10.8 mm.

**Female.** Differs from male as follows: *Head*: Frons 0.27–0.28 head width, upper and lower frons gradually widening ventrally, upper frons with slight medial depression in front of anterior ocellus, junction of upper and lower frons with indistinct elevation on each side of median line; upper frons with pale, appressed hair-like setae except on ocellar tubercle and in medial depression; antenna 3.6 times length of head; first five flagellomeres of antennal flagellum with dense, black velvety vestiture, with scattered fine, erect hair-like setae especially posteriorly, flagellomeres 6–8 with more erect, longer pilosity that is gradually longer toward apex, last flagellomere tapered apically; palpus with second segment more robust than in male.

*Thorax*: Scutum with hair-like setae mostly appressed, golden yellow; front femur sometimes more extensively dark on basal two-thirds; front tibia more extensively brownish black on dorsal part.

*Abdomen*: Tergite 2 and basal part of tergite 3 suffused with yellowish color medially; tergites 4–5 with shorter golden yellow hairs laterally and posterolaterally; sternite 8 with lateral margins evenly rounded, evenly continuous with posterior margin, produced dorsally along sides of terminalia; cercus yellowish brown, with first segment cylindrical, about three times as long as short, ovoid second segment.

*Length*: 10.3–10.4 mm.

#### Distribution.

Known from peninsular Malaysia and adjacent Thailand.

#### Type material examined.

The four syntypes noted in Brunetti (1923) are present in BMNH, three of which were subsequently labeled as syntypes. I am hereby designating the male specimen in the most complete condition as lectotype to stabilize the taxonomic concept of this species and its name. The specimen is labeled: “Siam: Bulsit Besar. H.C.Robinson & N.Annandale. 1916–21./LECTOTYPE ♂ *Parastratiosphecomyia stratiosphecomyioides* Brunetti, 1923 des. N. E. Woodley 2012”. As can be seen from the label data, this specimen had not previously been labeled as a syntype, but it clearly is one. There is no date on the data label. The specimen (Fig. 2) is in moderately good condition, but is missing the left antennal flagellum, the apical seven flagellomeres of the right antenna, and the right middle leg beyond the trochanter.

Lectotype male (BMNH), **THAILAND:** Pattani Province, Bukit Besar, H.C. Robinson and N. Annandale; paralectotype male (BMNH), same data but 2500 feet, 1.ix.1901; paralectotype male (BMNH), same data but 2500 feet, 30.viii.1901; paralectotype female (BMNH), same data but 2500 feet, 4.ix.1901.

####  Additional material examined.

**MALAYSIA:** 1 female (FSMU), Perak, Cameron Highlands, environs of Batu Village, 4°22'N, 101°20'E, 590 meters, v.2009, Pacholátko leg.

#### Remarks.

Brunetti (1923) cited the type locality for *Parastratiosphecomyia stratiosphecomyioides* as “Bukit Besar, Patani, Peninsular Siam”. However, the labels on all four of the original syntypes read “Bulsit Besar” rather than “Bukit”. Using internet searches I found nothing about a possible locality for Bulsit Besar, but there are several localities called Bukit Besar in peninsular Malaysia (it apparently means “Big Hill” in Malay). Although “Patani” is not part of the actual specimen labels, it seems that by some means Brunetti was aware that the type locality was in Patani. According to *Webster’s Geographical Dictionary* (Bethel 1967: 860) Patani was “formerly, a Malay state in the Malay Penin. under Siamese protection, included among the Malay States; now Pattani prov. in Thailand.” Still a province in southeastern Thailand, Pattani has some mountainous areas, so a locality of 2500 feet, as noted on data labels of some of the syntype specimens, is possible. Therefore, I think it is highly likely that the type locality is in Pattani province.

### Parastratiosphecomyia freidbergi

Woodley sp. n.

urn:lsid:zoobank.org:act:22EFA398-1323-45EB-B8CB-4943FEB5F033

http://species-id.net/wiki/Parastratiosphecomyia_freidbergi

Figs 1 , 3 , 7 , 10 , 16–20

#### Diagnosis.

*Parastratiosphecomyia freidbergi* can easily be distinguished from *Parastratiosphecomyia stratiosphecomyioides* because its antennal scape is not produced ventrally (Fig. 10) and its scutellum (Figs 1, 3) is more extensively black. It differs from *Parastratiosphecomyia rozkosnyi* and *Parastratiosphecomyia szechuanensis* by having the hind coxa uniformly pale, without any darkened areas, and by the structure of the male terminalia.

#### Description.

Differs from *Parastratiosphecomyia stratiosphecomyioides* as follows: **Male.** *Head*: Lower frons with dark spots larger and more distinct; face with a pair of nearly quadrate blackish spots below antennal bases, medial part not convex and only vaguely impressed medially, concave on lowest part between conical processes, medial portion shiny and only vaguely striate, conical process on lower, outer margin of antennal socket minute; pilosity on dark portion of lower face dark; antennal scape more evenly pilose, cylindrical, not produced ventrally, and with less evident concave area at inner base; second segment of palpus with basal half to entirely pale yellow.

*Thorax*: Scutum with pale spots at transverse suture more ovoid; scutellum with basal two-thirds to three-fourths blackish, with broad yellow margin; ventral part of katepisternum with mostly dark hair-like setae on dark cuticular region; front femur pale yellow at base, becoming brownish yellow on slightly less than apical half; middle femur pale yellow with distinct dark brown coloration on slightly less than apical half that is sharply delimited; hind femur entirely dark brown with extreme apex becoming yellowish; hind tibia almost completely dark brown, apicoventral third vaguely paler; wing with apical infuscation somewhat darker, the infuscation extending nearly to the wing apex, darkest area includes apical half of cell r_2+3_ and all of r_4_.

*Abdomen*: Medial portion of tergite 2 and basal portion of tergite 3 yellowish but the cuticular coloration largely obscured by dark pilosity; narrow lateral margins of tergites 2–5 distinctly yellow.

*Terminalia*: Gonocoxites (Fig. 16) with lateral margins nearly parallel, with a pair of ovoid dorsal processes that project posteriorly just anterior to gonocoxal apodemes and that have weakly serrate medial margins; posterior margin of synsternite with a pair of sharp, conical processes just ventral to gonostyli and a narrowly rounded medial process; gonocoxal apodemes just reaching anterior margin of genital capsule; gonostylus (Figs 16, 17) arcuate, with a large, subapical dorsal tooth and a small, narrowly rounded process just posterior to tooth; phallic complex (Figs 18, 19) larger than in *Parastratiosphecomyia stratiosphecomyioides*, more elongate, with medial lobe slightly longer than lateral lobes; epandrium (Fig. 20) large, evenly convex, posterior margin with rounded medial projection; epiproct and hypoproct deflexed ventrally.

*Length*: 11.3–12.0 mm.

**Female.** Unknown.

#### Distribution.

Known only from Meghalaya state in northeastern India.

#### Type material.

Holotype male (Fig. 3; USNM), **INDIA:** Meghalaya, Nongph [ = Nongpoh] Forest, 25–28.iv.1980, Amnon Freidberg. The holotype is in excellent condition. Paratype: 1 male (USNM), same data as holotype.

#### Etymology.

The species epithet, *friedbergi*, is a patronym in honor of Amnon Freidberg of Tel Aviv, Israel, whose excellent collecting over many years has produced numerous interesting Stratiomyidae.

#### Remarks.

This is the only species of *Parastratiosphecomyia* known from India and this represents the western-most record of the genus.

### Parastratiosphecomyia rozkosnyi

Woodley sp. n.

urn:lsid:zoobank.org:act:4AD5A769-C121-4B70-B6BC-F6F259B085CF

http://species-id.net/wiki/Parastratiosphecomyia_rozkosnyi

Figs 5 , 8 , 21–25

#### Diagnosis.

*Parastratiosphecomyia rozkosnyi* can be distinguished from *Parastratiosphecomyia stratiosphecomyioides* and *Parastratiosphecomyia freidbergi* by having the lateral surface of the hind coxa with some dark coloration. This can be small in extent but is always visible. It differs from *Parastratiosphecomyia szechuanensis* (as well as the other species) by its distinctive male genitalia in which the gonostylus possesses two subequal tooth-like processes (Fig. 22). Females are very similar to those of *Parastratiosphecomyia szechuanensis* but differ by having the juncture of the posterior and lateral margins of tergite 8 evenly rounded.

#### Description.

Differs from *Parastratiosphecomyia stratiosphecomyioides* as follows: **Male.** *Head*: Lower frons with dark spots larger and more distinct; face with a pair of irregularly ovoid blackish spots below antennal bases, medial part not convex and only vaguely impressed medially, concave on lowest part between conical processes, medial portion shiny and weakly to indistinctly striate, process on lower, outer margin of antennal socket minute, sometimes not developed; pilosity on dark portion of lower face dark; antennal scape more evenly pilose, sometimes darkened narrowly at base, slightly swollen, not produced ventrally, and concave area at inner base distinct but smaller.

*Thorax*: Scutum with lateral yellowish spots near transverse suture much smaller, not easily seen with naked eye, narrow ovoid; scutellum black with broad yellow posterior margin, the yellow coloration about one-fourth to one-third length of scutellum; scutum with hair-like setae entirely pale, golden yellow, with much of scutum also with scattered, more erect pale hair-like setae in addition to semi-appressed pilosity; hind coxa with some lateral darkened areas, sometimes extensive, the coloration somewhat diffuse rather than forming distinct markings; front femur brownish black on about basal one-third, this area sometimes yellowish dorsally, also a moderately well-defined brownish area ventrally near apex; middle femur coloration similar to front femur except that dark coloration at base occupies about half of the femur; hind femur brownish black becoming narrowly yellowish at apex; hind tibia brownish black, vaguely yellowish at extreme apex; tarsi with fifth tarsomeres brownish dorsally; wing with apical infuscation somewhat darker, the infuscation extending nearly to the wing apex, darkest area includes part of cell r_2+3_, all of r_4_, and basal three-fourths of r_5._

*Abdomen*: Tergite 1 brownish black with broad pale yellow margins both anteriorly and posteriorly, yellowish medially except for narrow band near base; tergite 2 yellowish medially, this coloration extending indistinctly on basal part of tergite 3.

*Terminalia*: Gonocoxites (Fig. 21) with lateral margins rounded, with a pair of posterodorsal processes that are rounded posteriorly, concave laterally; gonocoxal apodemes very small, ending far posterior of anterior margin of genital capsule; posterior margin of synsternite with narrow medial process that is rounded posteriorly; gonostylus (Figs 21, 22) arcuate, divided into a pair of sharp, subequal teeth; phallic complex (Figs 23, 24) small, narrow, trilobed, medial lobe subequal to lateral lobes in length; epandrium (Fig. 25) large, evenly convex, posterior margin with rounded medial projection; epiproct and hypoproct deflexed ventrally, hypoproct strongly sclerotized, not expanded dorsolaterally, with narrow anteromedial process that is slightly bent ventrally.

*Length*: 12.0–13.2 mm.

**Female.** Differs from male as follows: *Head*: Frons 0.25–0.27 head width, upper frons with slightly raised medial strip that is about one-fourth of head width and widens slightly toward antennae; dark spots above antennae small, sometimes indistinct; junction of upper and lower frons flat, face slightly concave medially; upper frons with pale, appressed hair-like setae.

*Thorax*: Scutum with erect hair-like setae slightly shorter than in male; hind tibia mostly brownish in specimens examined.

*Abdomen*: Sternite 8 with lateral margins extending dorsally toward posterior end, forming evenly rounded angle with posterior margin that does not overlap sides of terminalia.

*Length*: 10.3–10.4 mm.

#### Distribution.

Known from Laos and northern Thailand.

#### Type material.

Holotype male (Fig. 5; MMB), **LAOS:** Louang Namtha Province, Namtha to Muang Sing, 21°09'N, 101°19'E, 900–1200 m, 5–31.v.1997, Vít Kubán. Paratypes: 2 males, 1 female, same data as holotype; 1 male (MMB), **LAOS:** Oudomaxi Province, 17 km NEE of Oudom Xai, 20°45'N, 102°09'E, ca. 1100 m, 1–9.v.2002, Vít Kubán; 1 male (ZIB), **LAOS:** (central), environs of Ban Phabat, 70 km NE of Vientiane, 18°16.1'N, 103°10.9'E, 150 m, 27.iv–1.v.1997, E. Jendek, O. Sauša; 1 male, 1 female (SMF), **THAILAND:** (north), Mae Hong Son Province, Phangmapha, near Ban Nam Rin, 11.v.2011, D. Kovac, sweeping along small stream.

#### Etymology.

The species epithet, *rozkosnyi*, is a patronym in honor of Rudolf Rozkošný of Brno, Czech Republic, who has produced many excellent contributions to the knowledge of Stratiomyidae over a distinguished and continuing career.

#### Remarks.

*Parastratiosphecomyia rozkosnyi* is very similar to *Parastratiosphecomyia szechuanensis* in coloration and general structure. The main distinguishing features are the very different male terminalia and slightly different sternite 8 in the female.

**Figures 9–10. F5:**
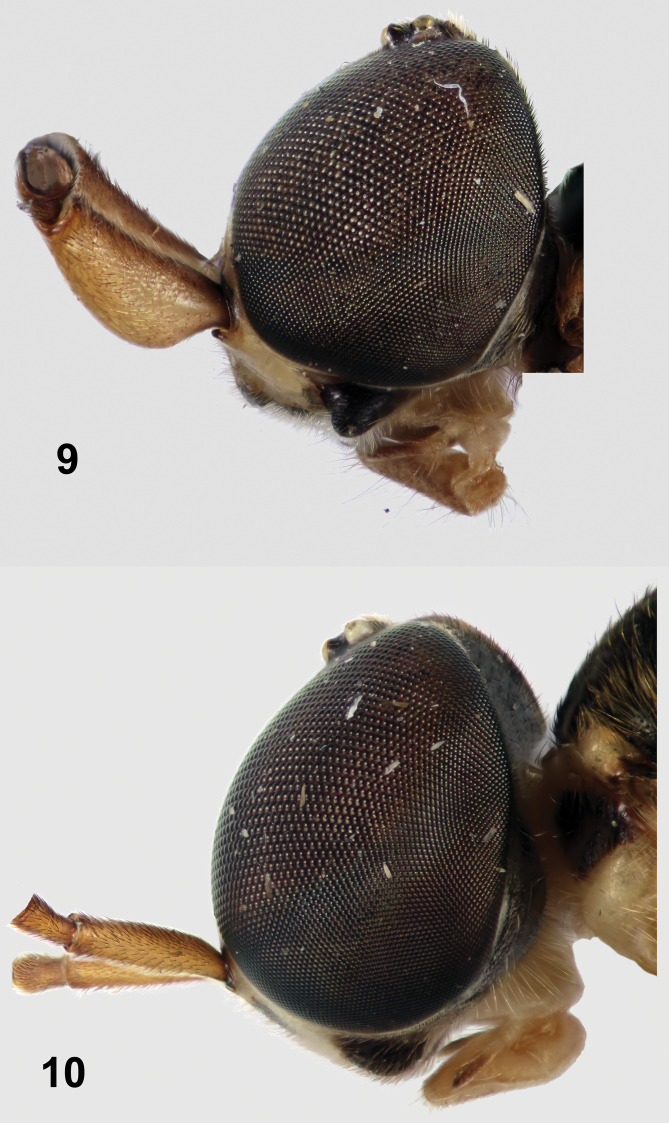
Left lateral views of heads of *Parastratiosphecomyia* species. **9** *Parastratiosphecomyia sphecomyioides* Brunetti **10** *Parastratiosphecomyia freidbergi* Woodley (antennal flagellae removed digitally).

**Figures 11–15. F6:**
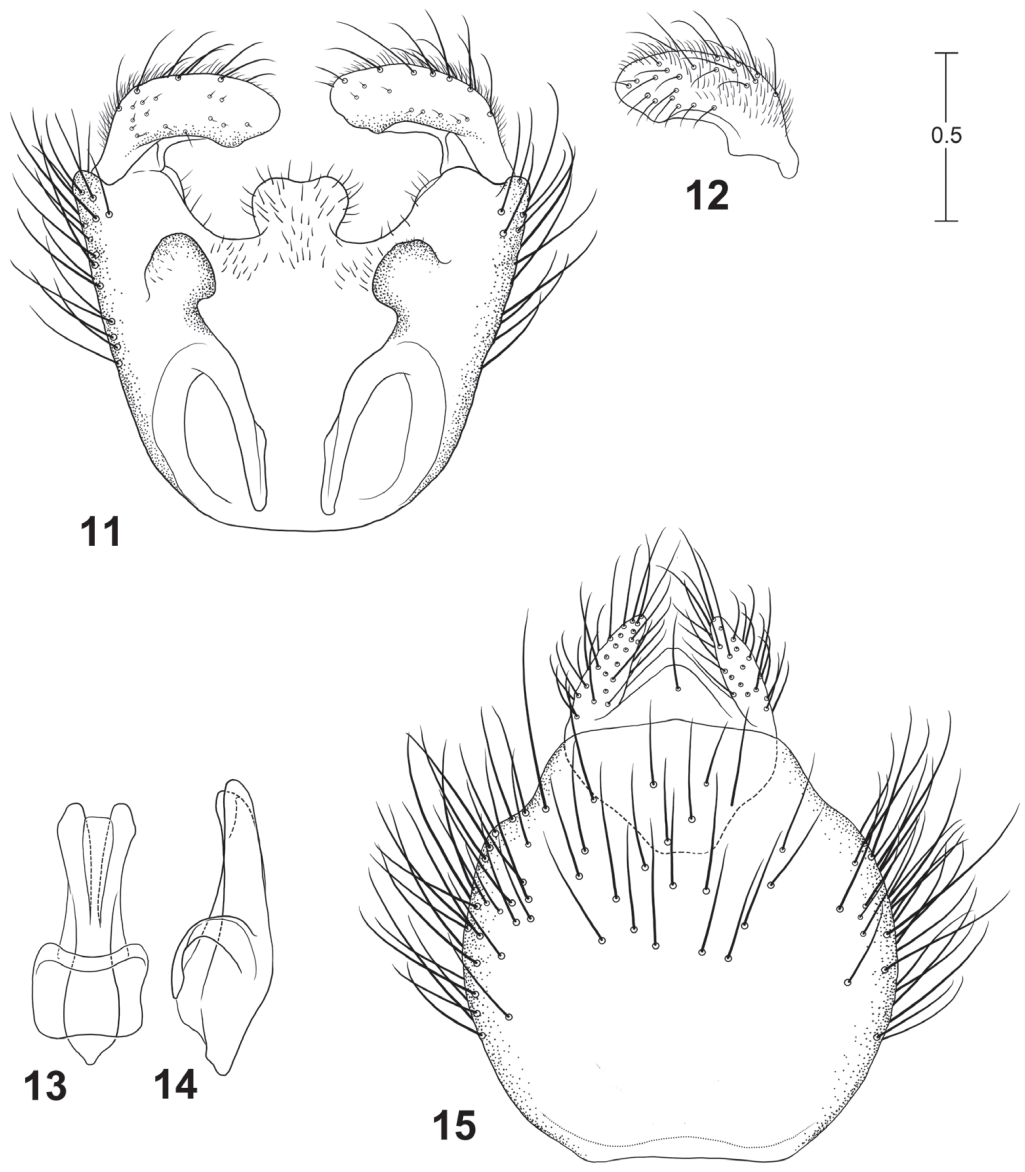
Male terminalia of *Parastratiosphecomyia sphecomyioides* Brunetti**. 11** Genital capsule, dorsal view **12** Gonostylus, anterolateral view **13** Phallic complex, dorsal view **14** Phallic complex, left lateral view **15** Epandrium and postgenital segments.

**Figures 16–20. F7:**
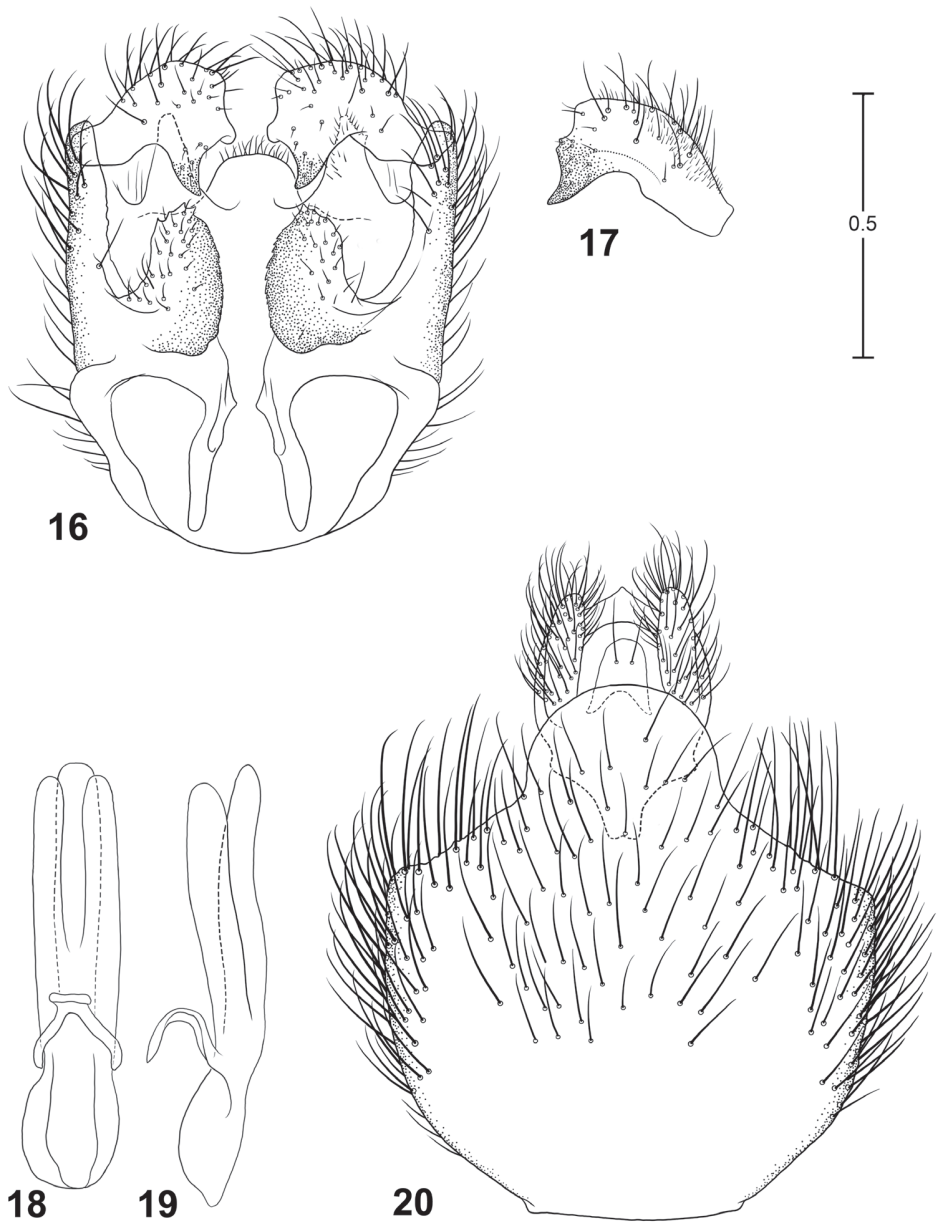
Male terminalia of *Parastratiosphecomyia freidbergi* Woodley. **16** Genital capsule, dorsal view **17** Gonostylus, lateral view **18** Phallic complex, dorsal view **19** Phallic complex, left lateral view **20** Epandrium and postgenital segments.

**Figures 21–25. F8:**
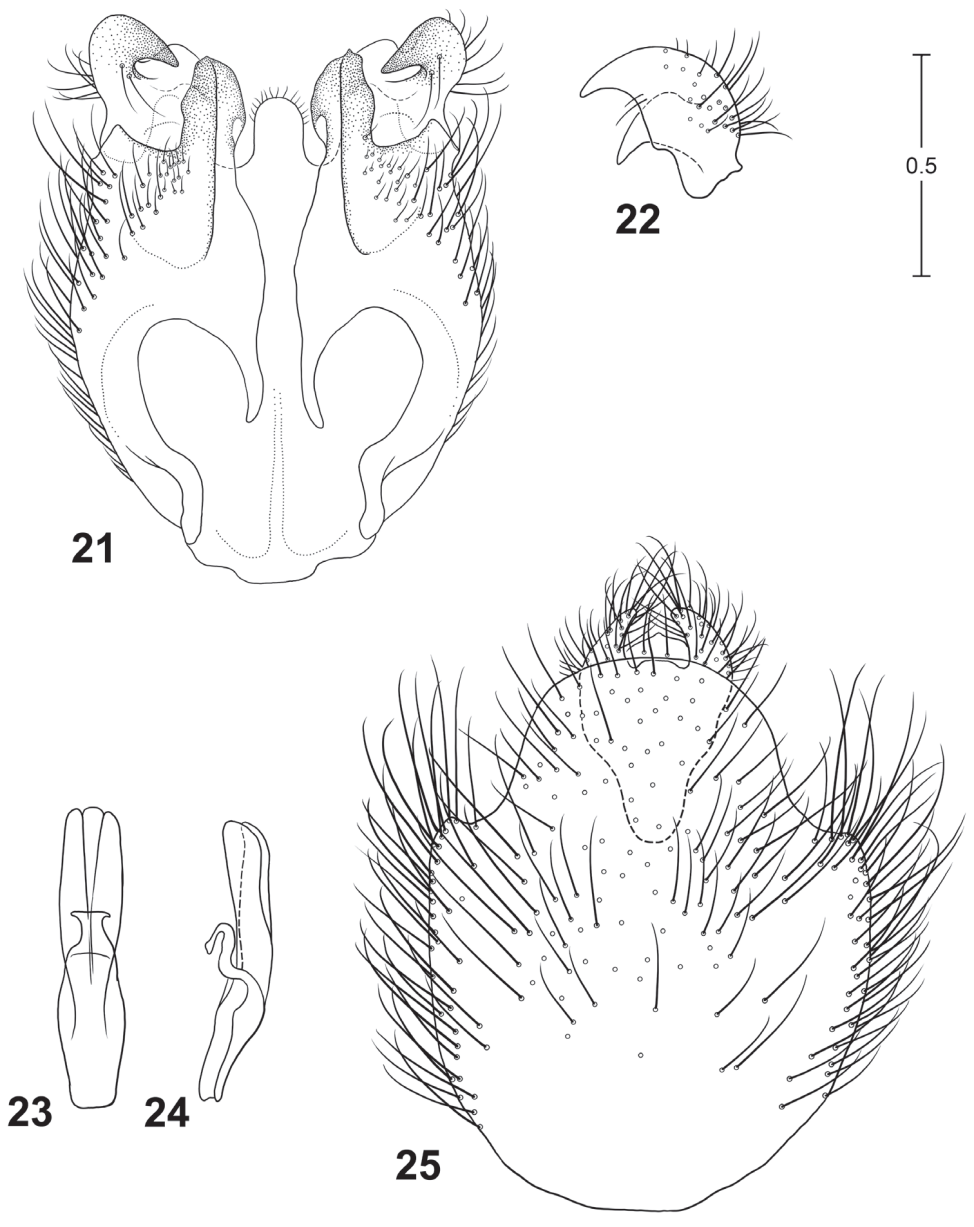
Male terminalia of *Parastratiosphecomyia rozkosnyi* Woodley. **21** Genital capsule, dorsal view **22** Gonostylus, anterolateral view **23** Phallic complex, dorsal view **24** Phallic complex, left lateral view **25** Epandrium and postgenital segments.

### Parastratiosphecomyia szechuanensis

Lindner, 1954

http://species-id.net/wiki/Parastratiosphecomyia_szechuanensis

Figs 4 , 26–30

Parastratiosphecomyia szechuanensis Lindner, 1954: 208.

#### Diagnosis. 

*Parastratiosphecomyia szechuanensis* can be distinguished from *Parastratiosphecomyia stratiosphecomyioides* and *Parastratiosphecomyia freidbergi* by having the lateral surface of the hind coxa with some dark coloration. This can be small in extent but is always visible. It differs from *Parastratiosphecomyia rozkosnyi* (as well as the other species) by its very distinctive male genitalia in which the gonostylus is elongate and sickle-shaped apically (Fig. 27). Females are very similar to those of *Parastratiosphecomyia rozkosnyi* but differ by having the juncture of the posterior and lateral margins of tergite 8 produced into a sharply rounded angle.

#### Redescription.

Differs from *Parastratiosphecomyia stratiosphecomyioides* as follows: **Male.** *Head*: Lower frons with dark spots larger and more distinct, sometimes taking up much of lower frontal surface; face with a pair of irregularly ovoid blackish spots below antennal bases, medial part not convex and only vaguely impressed medially, concave on lowest part between conical processes, medial portion shiny and only vaguely striate, conical process on lower, outer margin of antennal socket minute; pilosity on dark portion of lower face dark; antennal scape more evenly pilose, sometimes darkened narrowly at base, slightly swollen, not produced ventrally, and concave area at inner base distinct but smaller.

*Thorax*: Scutum with lateral yellowish spots near transverse suture much smaller, not easily seen with naked eye, narrow ovoid; scutellum black with broad yellow posterior margin, the yellow coloration about one-fourth to one-third length of scutellum; pleura with ventral part of meron sometimes only vaguely brownish; scutum with some dark hair-like setae on medial area, but much of scutum also with scattered, more erect pale hair-like setae in addition to semi-appressed pilosity; hind coxa with some lateral darkened areas, at least along posterolateral margin, the coloration somewhat diffuse rather than forming distinct markings; front femur with brownish coloration on basal one-half or less on ventral side but ranging to completely yellow, also a moderately well-defined brownish area ventrally near apex; middle femur coloration similar to front femur; hind femur ranging from basal three-fourths yellowish with apical one-fourth brownish black becoming slightly paler at apex, to having brownish coloration on basal part as well, but apical darkened area is always visible and darker than ground color; hind tibia usually almost entirely dark brown, but can be yellowish on up to apical three-fourths; tarsi with fifth tarsomeres brownish dorsally; wing with apical infuscation somewhat darker, the infuscation extending nearly to the wing apex, darkest area includes part of cell r_2+3_, all of r_4_, and basal three-fourths of r_5._

*Abdomen*: Tergite 1 brownish black with broad pale yellow margins both anteriorly and posteriorly, narrow medial region only vaguely or not yellowish; tergite 2 occasionally with indistinct yellowish coloration medially.

**Figures 26–30.**
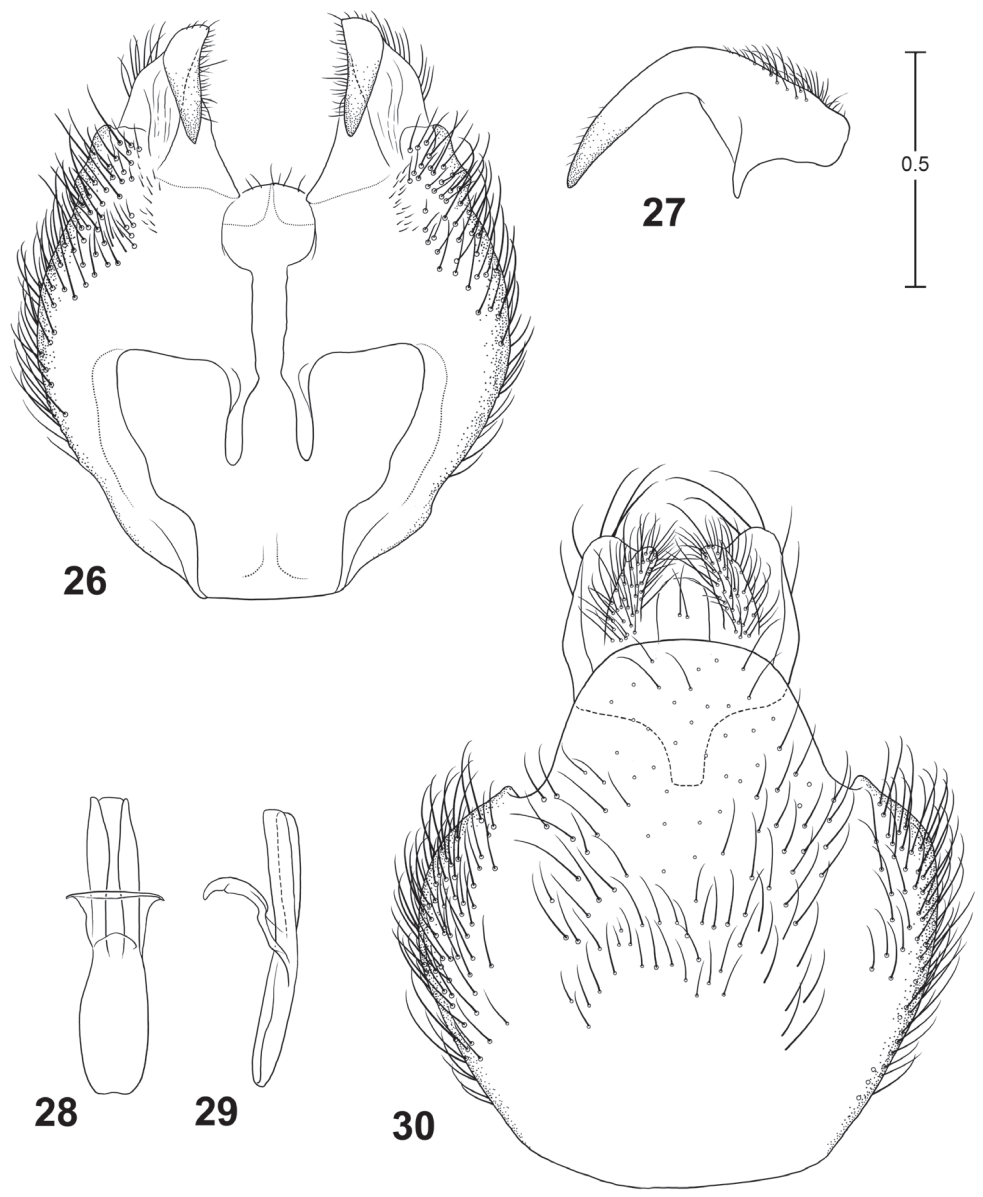
Male terminalia of *Parastratiosphecomyia freidbergi* Woodley. **26** Genital capsule, dorsal view **27** Gonostylus, anterolateral view **28** Phallic complex, dorsal view **29** Phallic complex, left lateral view **30** Epandrium and postgenital segments.

*Terminalia*: Gonocoxites (Fig. 26) with lateral margins rounded, with a pair of posterodorsal processes that are narrowly truncate posteriorly; gonocoxal apodemes very small, ending far posterior of anterior margin of genital capsule; posterior margin of synsternite rounded but mostly membranous, narrowly sclerotized medially which appears like a narrow process; gonostylus (Figs 26, 27) elongate, bent dorsally in medial region, appearing sickle-like in lateral view, with short, narrow basal tooth; phallic complex (Figs 28, 29) small, narrow, trilobed, medial lobe subequal to lateral lobes in length; epandrium (Fig. 30) large, evenly convex, posterior margin with rounded medial projection that is curved posteroventrally; epiproct and hypoproct deflexed ventrally, hypoproct strongly sclerotized, somewhat expanded dorsolaterally, with narrow anteromedial process that is bent ventrally.

*Length*: 12.0–13.2 mm.

**Female.** Differs from male as follows: *Head*: Frons 0.25–0.28 head width, upper frons with slightly raised medial strip that is about one-fourth of head width and widens slightly toward antennae; junction of upper and lower frons flat; upper frons with pale, appressed hair-like setae that are often sparse or nearly absent on medial elevation.

*Thorax*: Scutum with pilosity entirely pale, silvery to slightly golden, with scattered slightly longer, erect hair-like setae; hind tibia mostly brownish in specimens examined.

*Abdomen*: Tergite 2 with medial strip of yellowish coloration more distinct than in male, and basal part of tergite 3 suffused with yellowish color medially; sternite 8 with lateral margins extending dorsally toward posterior end, forming sharply rounded angle with posterior margin that is produced dorsally along sides of terminalia and slightly overlaps them.

*Length*: 11.3–13.6 mm.

#### Distribution. 

Known from China (Fujian, Guizhou), Laos, and Vietnam.

#### Type material examined.

The holotype male (Fig. 4; ZFMK) is labeled: “Kuatun(2300 m) 27,40n. Br. 117,40ö. L. J. Klapperich 5.6.1938(Fukien)/Parastratiospheco-myia szechua-nensis Lind. Lindner det./Holotypus Lindner 1954/Holotypus”. The specimen is in fair condition, missing the apical flagellomere of the right antenna, the left middle leg beyond trochanter, right middle tarsus, and the last tarsomere of the left hind leg, and the right halter. The abdomen has at sometime in the past been glued to the specimen and is slightly crooked. The specimen has a small amount of mold on it.

In ZFMK there are an additional 14 males and 4 females from the same locality that have later paratype labels not provided by Lindner. Of these, 4 males and all 4 females bear Lindner determination labels in his handwriting. Within this subset, 1 male and 2 females have paratype labels handwritten by Lindner, and 1 female is labeled as allotype in Lindner’s handwriting. Lindner (1954: 208) mentioned “eine größere Serie in beiden Geschlechtern” but no numbers, so it is not possible to determine if Lindner actually examined all of these specimens. He also mentioned that there were paratypes in the museum in Stuttgart, which I have not examined.

Specimens labeled as paratypes, all in ZFMK: 3 males, **CHINA:** Fujian Province, Guadun, 27°40'N, 117°40'E, “2300 m”, 5.v.1938, Klapperich; 1 male, same data but 13.v.1938; 1 male, same data but 4.vi.1938; 2 males, same data but 5.vi.1938; 3 males, same data but 8.vi.1938; 1 male, same data but 10.vi.1938; 1 female, same data but 12.vi.1938; 1 male, 1 female, same data but 17.vi.1938; 1 males, 2 females (1 labeled as allotype), same data but 26.vi.1938; 1 male, same data but 28.vi.1938.

#### Additional material examined.

**CHINA:** 1 male (MHPC), Guizhou Province, Xingyi, 800 m, 17.vii.2005, Yang Zaihua; 1 female (MHPC), Guizhou Province, Chishui, 315 m, 28.v.2006, Yang Zaihua. **LAOS:** 1 male (FSMU) Bolikhamsai Province, Ban Nape–Kaew Nua Pass, 18°22.3'N, 105°09.1'E (GPS), 600 ±100 m, 18.iv.–1.v.1998, E. Jendek, O. Sauša. **VIETNAM:** 1 female (NBC), Ninh Binh Province, Cuc Phuong National Park, near centre, ca. 225 m, 29.vi–18.vii.2000, Mai Phu Quy; 1 male, 2 females (USNM), [Ha Tay Province], Mount Ba Vi, 900–1000 m, viii.1940, P.A. de Cooman; 1 male (USNM), same data but 800–1000 m, vii.1941; 1 female (USNM), Cao Bang Province, Phja-Den environs, 22°32.433'N, 105°52.012'E, 948 m, 1–6.vi.2011, Steven W. Lingafelter, daytime collecting; 1 female (USNM), Cao Bang Province, Phja-Den environs, 22°34.026'N, 105°52.246'E, 987 m, 25.v.–5.vi.2011, Steven W. Lingafelter, at lights.

#### Remarks.

The type locality, as stated on the locality label, is “Kuatun, Fukien” which in modern lexicon is Guadun, Fujian Province, in China. This settlement is located in the Wuyi Mountains, west of Wuyishan city. The 2300 m elevation given on the data labels is inaccurate, as the highest point in the Wuyi Mountains is a peak that is about 2150 m, and it is likely that the specimens were collected at a lower elevation than this. The type locality is nearly 1000 km from Sichuan Province in China, so it is a mystery as to why Lindner named this species *Parastratiosphecomyia szechuanensis*.

Leg coloration in this species is somewhat variable, but this may be in part due to preservation and the age of the specimens when captured.

## Supplementary Material

XML Treatment for Parastratiosphecomyia

XML Treatment for Parastratiosphecomyia stratiosphecomyioides

XML Treatment for Parastratiosphecomyia freidbergi

XML Treatment for Parastratiosphecomyia rozkosnyi

XML Treatment for Parastratiosphecomyia szechuanensis
